# Return to Activity Following Isolated Ulnar Nerve Surgery: A Systematic Review

**DOI:** 10.7759/cureus.65854

**Published:** 2024-07-31

**Authors:** Xavier A Akins, Kashif Javid, Catherine M Will, Amy L Meyers, Austin V Stone

**Affiliations:** 1 Orthopaedic Surgery and Sports Medicine, University of Kentucky, Lexington, USA

**Keywords:** return to activity, decompression, transposition, ulnar nerve surgery, ulnar nerve

## Abstract

Ulnar neuropathy is one of the more commonly diagnosed mononeuropathies; despite this, a definitive surgical treatment strategy has not been widely agreed upon. In this study, we systematically review the literature and assess return to play or activity outcomes in patients with neuritis or neuropathy undergoing in situ decompression, subcutaneous transposition, or submuscular transposition of the ulnar nerve. We hypothesized that ulnar nerve transposition or decompression in the absence of concomitant ulnar collateral ligament (UCL) pathology would have a high rate of return to activity. Relevant studies were generated from 1975 to 2023 using PubMed, Academic Search Complete, CINAHL (Cumulative Index to Nursing and Allied Health Literature), MEDLINE, and SPORTDiscus. Articles reporting on return to play or activity outcomes following isolated ulnar nerve transposition or decompression for ulnar neuritis were included. Studies evaluating patients with concomitant UCL injury or revision surgery were excluded. A total of 12 studies met the inclusion criteria, ranging from 1977 to 2021. There were a total of 358 patients with a reported return to play or activity status across all studies with an average age of 27.2 years (range, 11-75). Successful return to play, activity, or work was reported in 303 patients (84.6%). Patients undergoing transposition, subcutaneous (n = 232) and submuscular (n = 20), had return rates of 87.9% and 95%, respectively. Patients undergoing in situ decompression (n = 106) had return rates of 75.5%. This systematic review found an 84.6% return to activity rate following ulnar nerve transposition or decompression in the absence of concomitant UCL pathology. Overall, transposition or decompression of the ulnar nerve provides a favorable return to activity rates and with appropriate indications and surgical technique will likely yield a successful return.

## Introduction and background

Ulnar neuropathy or neuritis due to cubital tunnel compression is a common problem faced by young and adult overhead athletes; despite this, a definitive surgical intervention has not been widely agreed upon. Although the injury commonly occurs in throwing athletes, it may also arise in musicians, military personnel, and other occupations. The presentation of ulnar neuropathy is classically described as numbness and tingling of the ulnar two digits and may be accompanied by motor weakness in severe cases. These symptoms may present acutely after an upper extremity trauma but commonly arise as overuse injuries with resulting chronic compression of the ulnar nerve. The repetitive overhead motion performed by a throwing athlete can exacerbate these symptoms. Compression of the ulnar nerve can present in isolation or may commonly occur in the setting of concomitant ulnar collateral ligament (UCL) injury [1].

**Figure 1:**
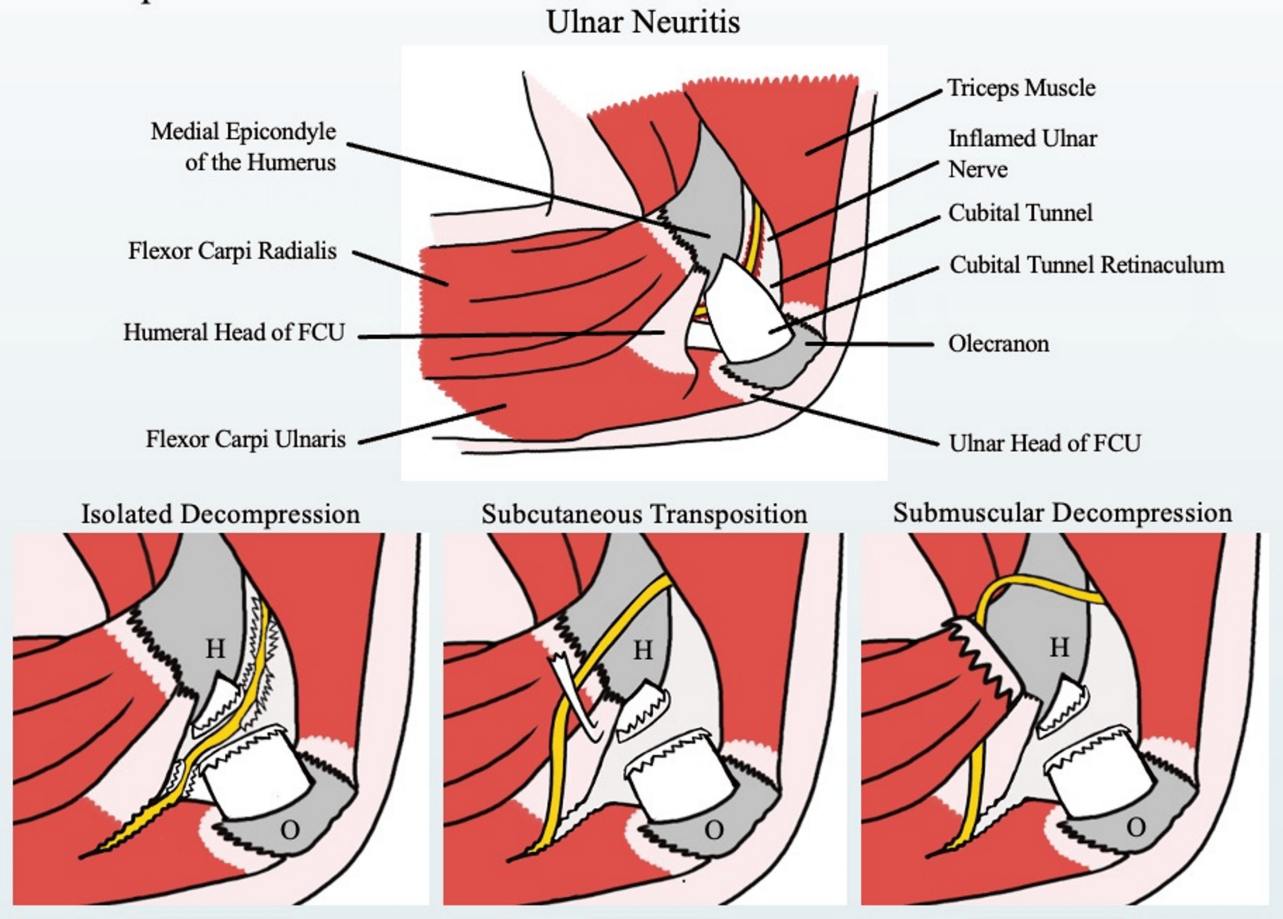
Surgical interventions for ulnar nerve compression. FCU: flexor carpi ulnaris; H: humerus; O: olecranon. The above illustration is the original work of the authors.

Treatment options for ulnar neuropathy include both non-operative and operative management. Non-operative management typically consists of activity modification, anti-inflammatories, and physical therapy. Regarding operative management, commonly used techniques include (1) in situ decompression, (2) subcutaneous transposition, and (3) submuscular transposition (Figure 1). The decision to pursue operative management should consider patients' desired level of activity, anatomy, and persistence of symptoms following less invasive surgical procedures. The surgeon should also be aware of the integrity of the UCL, as UCL incompetency is known to contribute to ulnar neuropathy in throwing athletes and often needs to be addressed to prevent recurrence [1].

Given the various treatment options available for ulnar neuropathy or neuritis, one should also consider the success rate of returning to play for the athlete after such procedures. Highly successful rates of return are reported for ulnar nerve transposition in the setting of ulnar collateral ligament reconstruction (UCLR); however, the success of return to activity with isolated ulnar nerve transposition is underreported. In this study, we systematically review the existing literature and evaluate outcomes related to return to activity in patients with ulnar neuritis or neuropathy who underwent ulnar nerve transposition or decompression with no concomitant UCL pathology. We hypothesize that isolated ulnar nerve transposition will have a high rate of return to activity.

## Review

Materials and methods

**Figure 2:**
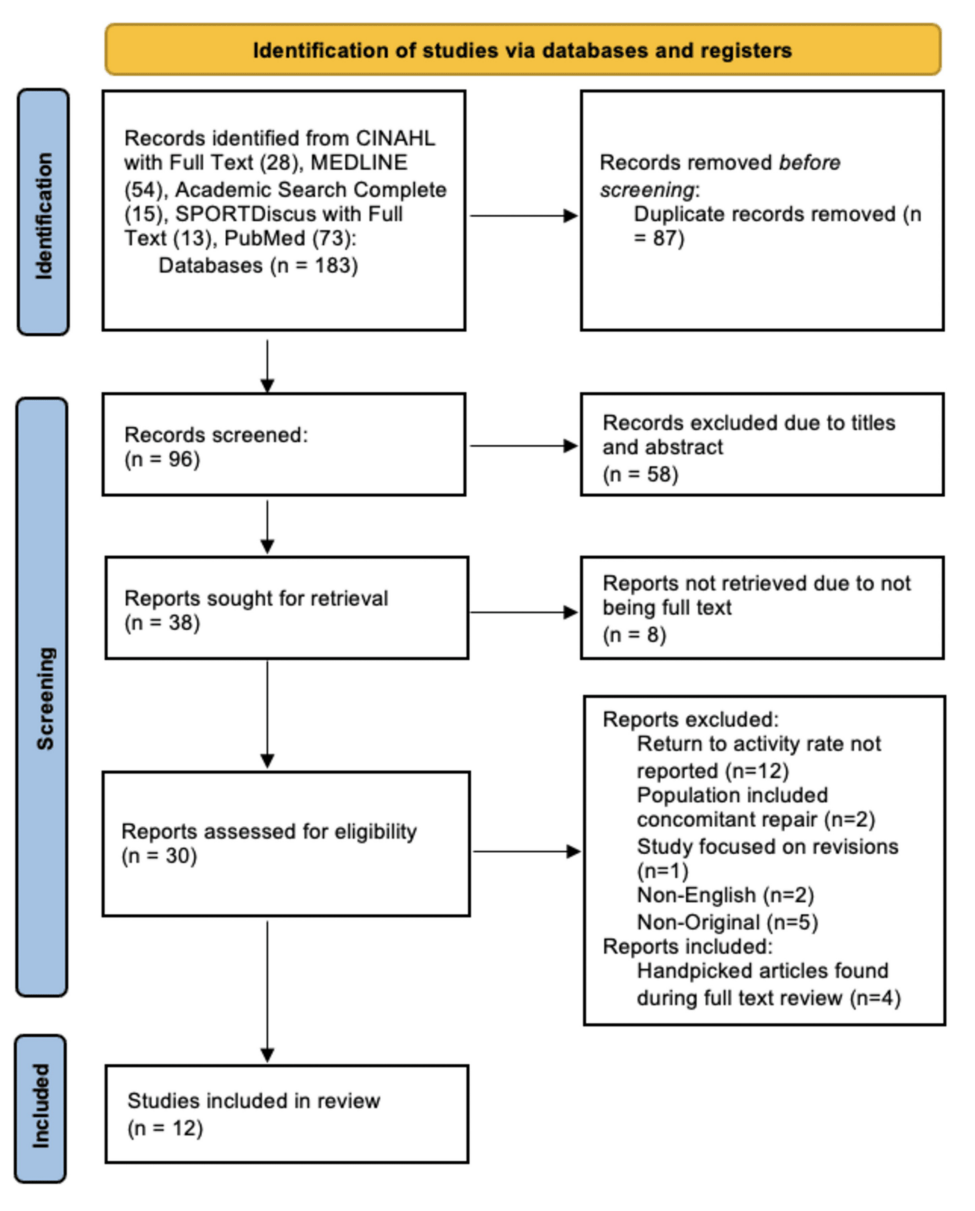
PRISMA flow diagram. PRISMA: Preferred Reporting Items for Systematic Reviews and Meta-Analyses.

A systematic review of the literature was done in accordance with the PRISMA (Preferred Reporting Items for Systematic Reviews and Meta-Analyses) guidelines (Figure 2) [2]. The goal of the search was to identify articles reporting on return to play or activity outcomes following isolated ulnar nerve transposition or decompression. The literature search was conducted using PubMed, Academic Search Complete, CINAHL (Cumulative Index to Nursing and Allied Health Literature), MEDLINE, and SPORTDiscus and included literature published between 1975 and 2021. Search terms were organized into the following search phrases to generate relevant articles: (ulnar nerve OR cubital tunnel) AND (transposition OR decompression) AND (return to sport OR return to play OR return to activity OR return to function). Relevant articles were then screened by two reviewers based on title and abstract. A preliminary review was followed by full-text screening considering inclusion and exclusion criteria. Articles were included if they reported (1) return to play or activity outcomes, (2) included ulnar neuritis or neuropathy, and (3) involved patients undergoing ulnar nerve transposition or decompression surgery. Studies were excluded if patients had (1) concomitant UCL pathology, or if (2) any previous surgery was done to address symptoms. Additionally, non-English and non-original studies were excluded from our analysis. Following full-text screening, 12 studies met inclusion criteria and were included in our analysis [3-14].To assess the quality of each article included, the Methodological Index for Non-Randomized Studies (MINORS) criteria was used [15]. This quality assessment tool is composed of 12 questions, with three of them addressing comparative studies. The global ideal score for comparative studies is 24 and the global ideal score for non-comparative studies is 16. The MINORS score was determined for each study by two reviewers who collaborated to establish a scoring consensus (Table 1).

**Table 1:** Methodological Index for Non-Randomized Studies (MINORS) quality assessment tool.

Quality assessment criteria	Hadley et al. (2021) [3]	Nicholson et al. (2020) [4]	Dunn et al. (2019) [14]	Smith et al. (2017) [5]	Aoki et al. (2005) [6]	Fitzgerald et al. (2004) [7]	Black et al. (2000) [8]	Rettig et al. (1993) [9]	Nathan et al. (1992) [10]	Richmond et al. (1982) [11]	Eaton et al. (1980) [12]	Del Pizzo et al. (1977) [13]
Clearly stated aim	2	2	2	1	2	2	2	2	1	2	2	1
Inclusion of consecutive patients	2	2	2	0	1	2	0	2	2	2	2	0
Prospective collection of data	2	2	2	2	0	0	0	2	0	0	0	0
Endpoints appropriate to the aim of the study	2	2	2	2	2	2	2	2	2	2	1	2
Unbiased assessment of study endpoint	0	0	0	0	0	0	0	0	0	0	0	0
Follow-up period appropriate to the aim of the study	2	2	2	1	2	2	2	2	2	2	2	2
Loss to follow up less than 5%	2	1	2	2	2	2	2	2	2	2	1	1
Prospective calculation of study size	0	0	2	0	0	0	2	0	0	0	0	1
Adequate control group	0	0	0	0	0	0	0	0	0	0	0	0
Contemporary groups	0	0	0	0	0	0	2	0	0	0	0	0
Baseline equivalence of groups	0	0	1	0	0	0	2	0	0	0	0	0
Adequate statistical analysis	0	2	2	0	0	1	0	0	0	0	0	0
Total score	12	13	17	8	9	11	14	12	9	10	8	7

Due to variation in sample sizes across the included studies, the statistical analysis included the use of weighted means to calculate the pooled return to activity rate. The percent of return to activity was taken from each study and multiplied by the proportion of the study sample size to the total patient population. These weighted values were then pooled together to generate a weighted mean. Subgroup analysis was then performed based on the surgical technique described within each study, with weighted means based on the study sample size calculated for each technique through the method described above.

Results

A total of 12 studies met inclusion criteria with a reported 358 patients (Table 2). The 12 studies included reported the use of three surgical techniques, with nine studies evaluating subcutaneous transposition/transfer, two reporting in situ decompression, and one analyzing submuscular transposition. One study compared subcutaneous transposition and simple decompression and had two populations as a result. The 12 included studies reported on a variety of physical activities that participants were aiming to return to function in, including amateur to professional sports, playing instruments, military duty, and other occupations. Overall, the return to activity rate across all studies evaluating isolated ulnar nerve surgery was 84.6% when considering individual sample size.

**Table 2:** Study and patient characteristics.

Study	Study design	Mean age (range)	Patient No.	Surgery No.	Outcome metric
Hadley et al. (2021) [3]	Retrospective	19.2 (15.6-28.0)	15	-	Return to play
Nicholson et al. (2020) [4]	Retrospective	18.4 (11-25)	26	-	Return to sport
Dunn et al. (2019) [14]	Retrospective	32.3	Simple decompression (65), subcutaneous transposition (67)	-	Remain in the military
Smith et al. (2017) [5]	Prospective	18 (16-20)	6	-	Return to sport
Aoki et al. (2005) [6]	Case series	14 (12-16)	6	-	Return to sport
Fitzgerald et al. (2004) [7]	Retrospective	34 (28-42)	20	-	Return to active duty
Black et al. (2000) [8]	Retrospective cohort	39.9 (17-68)	47	51	Return to work
Rettig et al. (1993) [9]	Retrospective	22 (13-53)	20	21	Return to sport
Nathan et al. (1992) [10]	Prospective cohort	42.5	41	52	Return to work
Richmond et al. (1982) [11]	Prospective cohort	32 (13-72)	16	18	Return to activity
Eaton et al. (1980) [12]	Prospective cohort	36 (18-75)	14	15	Return to play and activity
Del Pizzo et al. (1977) [13]	Prospective cohort	(17-31)	19	20	Return to play

Subcutaneous transposition/transfer (Table 3) had a weighted mean return to activity rate of 87.9%, with a total of 204 of 232 reportedly returning to activity, work, or sport. Of the 10 studies included, four reported on the level of performance subjects returned to and found that on average 67.9% returned to the same or higher level of performance following their procedure. This procedure has been well studied over time, with the study range being 1977-2021.

**Table 3:** Subcutaneous transposition return to activity rate. RTA: return to activity; SAT: subcutaneous anterior transposition.

Study	Sample size (N)	RTA (No.)	Percent	Same or higher level of return (No.)	Percent return to same level or higher
Eaton et al. (1980) [12]	14	13	92.9	-	-
Aoki et al. (2005) [6]	6	5	83.3	5	83.3
Dunn (SAT) et al. (2019) [14]	67	56	84	-	-
Black et al. (2000) [8]	47	46	97.9	-	-
Richmond et al. (1982) [11]	16	14	87.5	-	-
Hadley et al. (2021) [3]	15	13	86.7	10	66.7
Smith et al. (2017) [5]	6	5	83.3	5	83.3
Nicholson et al. (2020) [4]	26	24	92	16	62
Del Pizzo et al. (1977) [13]	15	9	60	-	-
Rettig et al. (1993) [9]	20	19	95	-	-
Total	232	204	87.9	36	67.9

Fewer studies reported a return to activity following ulnar nerve decompression (Table 4), with 106 patients included across two studies. Of these 106 patients, 80 returned to activity, representing a weighted mean return rate of 75.5%. No study reported the level of performance subjects returned to after their procedures. Dunn et al. [14] directly compared in situ decompression and anterior subcutaneous transposition and found that in situ decompression had a lower return to activity rate of 77% compared to 84% of patients who underwent subcutaneous anterior transposition. However, no statistically significant difference between the two procedures was found in the ability to return and remain in active duty in the military at two years follow-up (p = 0.39) [14].

**Table 4:** Simple decompression return to activity rate. RTA: return to activity; SD: simple decompression.

Study	Sample size (N)	Number to RTA	Percent to RTA (%)
Nathan et al. (1992) [10]	41	30	73
Dunn (SD) et al. (2019) [14]	65	50	77
Total	106	80	75.5

One study reported on submuscular transposition (Table 5) and found that 95% of participants were able to return to activity [7]. However, the sample size of this study was 20, limiting extrapolation of its findings when compared to the other surgical techniques evaluated in our study with sample sizes of 106 and 205 subjects, respectively. Of the studies included in our analysis, seven reported reasons for lack of return to activity for 10 patients. Five patients had persistent dysesthesia, paresthesia, or limitation that prevented a return to activity. Two patients were unable to return due to preexisting neuropathy (chronic diabetic neuropathy and McGowan grade 3 neuropathy). Two additional patients were unable to return to sport due to reinjury or other injuries and one patient did not return due to a loss of interest [3,5].

**Table 5:** Submuscular transposition return to activity rate. RTA: return to activity.

Study	Sample size (N)	Number to RTA	Percent to RTA (%)
Fitzgerald et al. (2004) [7]	20	19	95.0
Total	20	19	95.0

Discussion

The principal finding of our study is that isolated ulnar nerve surgery in patients with refractory ulnar neuritis is associated with favorable postoperative outcomes and a high rate of return to activity, work, and sports. Patients can expect successful outcomes greater than 75% of the time following isolated ulnar nerve surgery regardless of the technique used when performed for the proper indications. While we were unable to stratify results based on type and level of function returning to due to a lack of consistent reporting, our overall weighted mean return to activity rate represented a favorable conclusion on the value of isolated ulnar surgery. Our data did show that subcutaneous transposition may be the preferred technique given its frequency, well-studied outcomes, and efficacy in return to play when compared to a lower rate of return for patients undergoing in situ decompression, and the sparsely studied submuscular transposition in active patients. Ulnar neuritis is initially managed non-operatively with surgical intervention considered in the setting of failed non-operative management or the presence of additional neurologic deficits [16]. The decision to pursue one surgical treatment option over another is controversial and surgeon-dependent. Additional factors clinicians should consider include the etiology of the neuritis, the severity of symptoms, and the presence or absence of subluxation of the ulnar nerve.

Simple or In Situ Decompression

Simple or in situ decompression is the treatment of choice in the absence of anatomic lesions and has proven to be an effective treatment option without the risk of compromising the vascularity of the ulnar nerve [16,17]. Two cohort studies reporting on return to play or activity following decompression were included in our study. Of the 106 patients that underwent in situ decompression, 80 returned to play or activity (75.7%). Whether or not the patients returned to the same level or higher was not reported. The favorable return to play or activity rate following ulnar nerve decompression found in our study is in accordance with other studies that also report positive postoperative outcomes following this procedure. In a randomized control trial comparing isolated decompression to subcutaneous transposition, Bartels et al. [18] found a complication rate of 9.6% for in situ decompression compared to 31.1% for subcutaneous transposition. The simplicity of the procedure and fewer associated complications may result in surgeons electing to employ this approach prior to exploring other surgical options [18]. Although this procedure has relatively positive postoperative outcomes and fewer procedural complications, surgeons may want to explore transposition. Staples et al. [19] previously reported that in situ decompression was a successful intervention for patients with a stable ulnar nerve; however, it is associated with a higher incidence of recurrent neuritis and subsequent revision in comparison to anterior transposition. This finding was further demonstrated in a retrospective cohort study by Hutchinson et al. [20] that reported a revision rate of 25% following in situ decompression compared to 12% for subcutaneous transposition.

Subcutaneous Transposition

To decrease the risk of recurrence, surgeons may elect to pursue subcutaneous transposition as their preferred treatment of choice. The ulnar nerve can be compressed at any point along its nerve course, anterior transposition addresses this and ensures adequate relief of compression [16]. Further indications for this technique include complicated cases of neuropathy with accompanying bony abnormalities, trauma, and prolonged duration of severe symptoms. Our analysis included 10 studies reporting on return to play or activity outcomes following subcutaneous transposition of the ulnar nerve. Of the 232 patients managed with this surgical technique, 204 returned to play or activity (87.9%). Additionally, 67.9% returned to the same level of play or higher. This suggests that subcutaneous transposition is a suitable treatment option for patients who are motivated to return to their baseline level of activity. These findings are comparable to previous studies also reporting positive outcomes following subcutaneous transposition [16,21,22]. Jaddue et al. [21] further reported the utility of the subcutaneous approach and found that it was associated with shorter incisions, decreased operative time, less postoperative pain, and fewer complications than submuscular transposition. Although subcutaneous transposition has proven to be an effective intervention, proponents of submuscular transposition caution against the use of a subcutaneous approach as thinner patients may have a predisposition to repeated minor trauma if the nerve is transposed subcutaneously [16].

Submuscular Transposition

In the event of chronic or more severe neuropathy with accompanying muscular atrophy, submuscular transposition is often considered [16]. Additional uses of the submuscular approach include thinner patient populations or revisions following other failed operative interventions [22]. In our study, submuscular transposition was found to have a return to play or activity rate of 95%. These findings are in accordance with Zarezadeh et al. [23], who reported favorable outcomes in cases managed with submuscular transposition with respect to decreased postoperative pain. The highest rate of return to play or activity was found in our study following submuscular transposition; however, this finding is not without limitations. Fitzgerald et al. [7] was the only study in our review that evaluated return to activity following this technique. The smaller sample size (n = 20) limits the statistical significance and external validity of our findings and calls for more studies that evaluate return to play outcomes following this technique. Although submuscular transposition was found to have favorable postoperative outcomes, it is commonly reserved for revision purposes given its invasive nature and extensive dissection of the flexor muscles [24]. The return to play or activity rates in our study are comparable to rates of return following other upper extremity surgical interventions. Erickson et al. [25] previously evaluated return to sport following UCLR in Major League Baseball (MLB) players and observed a return rate of 83%. Similar outcomes are also seen in patients undergoing surgery for neurogenic thoracic outlet syndrome (NTOS) secondary to the repetitive motion of the upper extremity. Shutze et al. [26] reported a return rate of 70% following surgery for NTOS.

## Conclusions

Despite the relative indications for each surgical technique, the literature is scarce on head-to-head trials that definitively favor one technique over another. Future research should aim to further stratify return to play or activity and performance following isolated ulnar nerve surgery as well as evaluate pre-operative risk factors that may impact the rate of return. Overall, isolated ulnar nerve surgery for refractory ulnar neuritis provides a favorable return to activity rate, and with appropriate indications and surgical technique will likely predict a successful return to activity.
